# Semen cryopreservation in men undergoing cancer chemotherapy--a UK survey.

**DOI:** 10.1038/bjc.1989.399

**Published:** 1989-12

**Authors:** D. W. Milligan, R. Hughes, K. S. Lindsay

**Affiliations:** Department of Haematology, East Birmingham Hospital, UK.

## Abstract

A questionnaire was sent to all centres in the UK involved in cryopreservation of semen from men with malignant disease. Details were requested of samples stored from 1977 to 1987 and of successful use of this sperm in subsequently achieving pregnancy. Twenty-two centres are involved and have stored specimens from 2,219 men. There has been a three-fold increase in referrals in the past five years. Three regions in England have no service. Twenty-seven pregnancies (21 live births) have been achieved in 22 couples. Laboratories are poorly funded, making adequate record keeping and audit of the service difficult.


*Br. J. Cancer* (1989), **60**, 966–967 © The Macmillan Press Ltd., 1989

### Semen cryopreservation in men undergoing cancer chemotherapy – a UK survey

D.W. Milligan[1], R. Hughes[1] & K.S. Lindsay[2]

[1]*Department of Haematology, East Birmingham Hospital, Birmingham B9 5ST, UK; and* [2]*Reproductive Medicine Unit, Queen Charlottes and Chelsea Hospital, London W6 0X9, UK.*

**Summary** A questionnaire was sent to all centres in the UK involved in cryopreservation of semen from men with malignant disease. Details were requested of samples stored from 1977 to 1987 and of successful use of this sperm in subsequently achieving pregnancy. Twenty-two centres are involved and have stored specimens from 2,219 men. There has been a three-fold increase in referrals in the past five years. Three regions in England have no service. Twenty-seven pregnancies (21 live births) have been achieved in 22 couples. Laboratories are poorly funded, making adequate record keeping and audit of the service difficult.

The price that many young men pay for the curative treatment of malignant disease is infertility. It has been appreciated for many years that anti-cancer treatment may result in azoospermia (Sutcliffe, 1987), which is frequently permanent (Da Cunha *et al.*, 1984). The damage to the testes depends partly on the choice of drug – alkylating agents do the most harm (Roeser *et al.*, 1978; Miller, 1971) – and partly on the duration of exposure (Waxman, 1985). Damage is mainly confined to the germinal epithelium and Leydig cell function is normal. This causes infertility but preserves libido and potency.

The success of modern treatments for Hodgkin's disease, non-Hodgkin's lymphoma and leukaemia has resulted in increasing numbers of men who survive but who are infertile. Some schedules for advanced germ cell tumours may also cause infertility. Previous studies (Hendry *et al.*, 1983) have demonstrated that insemination with semen cryopreserved before the start of chemotherapy may result in successful pregnancy. Unfortunately many of these patients present with low sperm counts or poorly motile sperm before treatment starts (Sanger *et al.*, 1980). Despite these limitations a number of centres in the UK offer semen cryopreservation for cancer patients. Although a single centre has published its results (Hendry *et al.*, 1983; Scammell *et al.*, 1985) the practice throughout the UK has not been reviewed. We set out to examine the availability of this service throughout the country and to assess its success.

### Methods

Postal questionnaires were sent to clinics and hospitals throughout England, Wales, Scotland and Northern Ireland notified to the Fertility Sub-committee of the Royal College of Obstetricians and Gynaecologists. Some centres were not recorded by this committee and questionnaires were sent to them after informal enquiry. Where necessary, additional information was obtained by telephone or further correspondence. Respondents were asked to detail their experience of semen cryopreservation in the period 1977–87. Details were requested of the number of samples stored, the sperm counts and motility and the underlying diagnosis. Information about the success of stored semen in achieving pregnancy from 1977 to the present day was sought and in those instances where pregnancy occurred, details of sperm quality and insemination techniques were requested.

### Results

The response was 100%. Twenty-two centres in the UK have cryopreserved semen from 2,219 men with malignant disease (Table I). Hospitals vary widely in the number of patients referred, the largest centres seeing five per month on average and the smallest an occasional patient only. Over the past 5 years there has been a considerable increase in activity, the number of new patients referred almost trebling when compared with the first 5 years of the study. There was a wide geographical difference in the availability of this service throughout the country. Some regions (Oxford, Mersey and Yorkshire) have no service and in East Anglia the service is provided by the private sector but paid for by the NHS. Facilities were provided roughly equally between NHS and university departments. Although not specifically requested, information gained informally suggested that specific funding for this area was not set aside from Regional or District Health Authority budgets. Many hospitals did not keep accurate records of the primary diagnosis of the men referred but when this was available the majority of the patients had either germ cell tumours or advanced Hodgkin's disease. Not all hospitals recorded when specimens with a low sperm count or poor motility were rejected for storage but on average 27% (range 0–53%) were considered unsuitable for cryopreservation.

### *Artificial insemination*

Twelve of the 22 hospitals have used frozen semen for artifical insemination in 133 couples. Five of these 12 centres have achieved successful pregnancies. There have been 27 pregnancies in 23 couples; these have ended in miscarriage on six occasions and 21 normal deliveries. Further data were collected on 18 of the pregnancies ending in live births. Insemination was intracervical in all but two cases where GIFT was used. The mean post-thaw sperm count was $55 \times 10^6 \, ml^{-1}$ and the mean sperm motility was 39%. The

**Table I** Cryopreserved semen from men with malignant disease in the United Kingdom 1977–87

| *Region* | *No. stored* | *Attempted conception (couples)* | *Pregnancies* | *Live births* |
|---|---|---|---|---|
| Scotland | 104 | 10 | 0 | 0 |
| Northern Ireland | 18 | 1 | 0 | 0 |
| Wales | 31 | 3 | 0 | 0 |
| London[a] | 922 | 51 | 14 | 10 |
| Northern | 37 | 0 | 0 | 0 |
| Trent | 360 | 29 | 3 | 2 |
| West Midlands | 88 | 3 | 0 | 0 |
| North Western | 419 | 21 | 8 | 7 |
| East Anglia | 18 | 0 | 0 | 0 |
| South West | 172 | 13 | 2 | 2 |
| Wessex | 50 | 2 | 0 | 0 |
| Total | 2219 | 133 | 27 | 21 |

[a] All Thames regions.

Correspondence: D.W. Milligan.
Received 11 May 1989; and in revised form 31 July 1989.

lowest sperm count which achieved successful pregnancy was $10 \times 10^6 ml^{-1}$ and the lowest motility was 20%. On average 3.4 cycles were needed to achieve pregnancy. Hormone monitoring of the female cycle was used in all but three of the 24 pregnancies where this information was available. In two couples pregnancy was attempted by IVF but after successful fertilisation of the ova there was failure of implantation. The mean sperm storage time before successful pregnancy was 47 months, the maximum being 113 months.

### Discussion

Despite the 100% response to the questionnaire the quality of the information provided was frequently poor and the results obtained can only offer a broad overview of current practice in this country. We do feel that this is an important area of debate and one that is all too easily overlooked in the rush to confirm a diagnosis and start treatment. The development of infertility is a heavy blow to many men undergoing cytotoxic therapy for malignant disease. As the outlook for some cancers has improved, the number of young patients with infertility as a permanent consequence of treatment has increased. Many doctors find this a difficult area to discuss, especially when the patient is already burdened with the impact of the primary diagnosis and the prospect of chemotherapy. Frequently patients are too sick at presentation to permit collection of semen and there is a natural tendency for the oncologists to wish to press on with treatment as soon as possible. A common observation of respondents to the questionnaire was that patients were referred for semen storage rather as an afterthought and inadequate time was allowed for semen collection.

This survey was conducted with a view to defining the service that exists throughout the country but also to examine which specimens were worth storing. Successful pregnancies have generally been achieved using sperm with a normal count and good motility. The majority of pregnancies have been aided by female hormone monitoring. The development of new techniques of assisted conception (IVF and GIFT) may allow successful pregnancy using semen of poorer quality and at present it is difficult to define sperm counts below which storage is not worthwhile but a realistic target would be the presence of $5 \times 10^6$ motile sperm per ml. This figure will need to be reviewed as skills in this area increase. We consider that in order to avoid possible future litigation semen cryopreservation should be offered to all patients fulfilling these criteria when possible. The guidelines recently suggested are very sensible (Selby *et al.*, 1988). The same authors have also highlighted the problems that may be caused by the government white paper (Anon, 1987) on the disposal of stored sperm. This suggests a maximum storage time of 10 years. In this series two pregnancies occurred after a storage time of 113 months and in future it is likely that men will wish to use sperm that has been cryopreserved longer than this. The Warnock Committee proposal of regular review of all stored samples at 5-year intervals would accommodate late conceptions but with adequate safeguards.

Overall the achievement of 21 live births when specimens have been stored from in excess of 2,000 patients may seem small. However, only 133 couples have been referred for insemination. In the five hospitals where pregnancies occurred, about one-quarter of referred couples achieved a pregnancy. It was clear from analysis of the returns that many laboratories had been established without specific funding to carry out this work. As the referrals have increased it has proved increasingly difficult to keep adequate records. Only two departments had computerised record keeping. Many laboratories had no formal system of identifying which patients had died. Problems with record keeping also extended to inadequate feedback information on the success or otherwise of attempts at artificial insemination. For instance, one large laboratory was unable to provide data on conceptions although it had stored 20% of the samples in the UK.

In conclusion, it appears that semen cryopreservation is effective for some young men with malignant disease. Adequate funding and staffing of the departments undertaking this work would allow for a much more effective audit of the results, which would simplify exclusion criteria for sperm storage and define optimum techniques of achieving pregnancy.

We are most grateful to the following for completion of the questionnaire and for permission to publish data from their laboratories: L. Bell, Aberdeen Royal Infirmary; Professor J. Newton, J. Cuthbert, Birmingham Maternity Hospital; R. Simons, Bourne Hall Clinic, Cambridge; E. Crosier, BUPA, Norwich; J. Crich, City Hospital, Nottingham; Dr D. Richardson, MRC Reproductive Biology Unit, Edinburgh; Professor Sir Malcolm MacNaughton, W. McNally, Glasgow Royal Infirmary; Dr B. Mason, S. Avery, Hallam Clinic, London; Dr C. Barratt, Jessop Hospital for Women, Sheffield; C.R. Stewart, Leicester General Hospital; Dr G. Bahadur, Middlesex Hospital, London; J. Mills, Ninewells Hospital, Dundee; D.W. Sykes, Dr B. Pepper, Royal Devon and Exeter Hospital; Professor R. Shaw, Royal Free Hospital, London; Professor W. Thompson, Royal Maternity Hospital, Belfast; Dr E.H.E. Pease, A. Atkinson, St Mary's Hospital, Manchester; G.M. Masson, B. Purdie, Princess Anne Hospital, Southampton; D. Joyce, T. Jones, Southmead Hospital, Bristol; Dr D.K. Goff, Royal Infirmary, Sunderland; Dr B. Bean, Watford General Hospital; S.M. Walker, A. Frazer, University Hospital of Wales, Cardiff.

### References

ANON (1987). Human fertilisations and embryology: a framework for legislation (CM 259). HM Stationery Office: London.

DA CUNHA, M.F., MEISTRICH, M.L., FULLER, L.M. & 7 others (1984). Recovery of spermatogenesis after treatment for Hodgkin's disease. *J. Clin. Oncol.*, **2**, 571.

HENDRY, W.F., STEDRONSKA, J., JONES, C.R., BLACKMORE, C.A., BARRETT, A. & PECKMAN, M.J. (1983). Semen analysis in testicular cancer and Hodgkin's disease: pre- and post-treatment findings and implications for cryopreservation. *Br. J. Urol.*, **55**, 769.

MILLER, D.G. (1971). Alkylating agents and human spermatogenesis. *JAMA*, **217**, 1662.

ROESER, H.P., STOCKS, A.E. & SMITH, A.J. (1978). Testicular damage due to cytotoxic drugs and recovery after cessation of therapy. *Aust. NZ J. Med.*, **8**, 250.

SANGER, W.G., ARMITAGE, J.O. & SCHMIDT, M.A. (1980). Feasibility of semen cryopreservation in patients with malignant disease. *JAMA*, **244**, 789.

SCAMMELL, G.E., STEDRONSKA, J., EDMONDS, D.K., WHITE, N., HENDRY, W.F. & JEFFCOATE, S.L. (1985). Cryopreservation of semen in men with testicular tumour or Hodgkin's disease. Results of artificial insemination of their partners. *Lancet*, **ii**, 31.

SELBY, P., BRADA, M., HORWICH, A., WILTSHAW, E., McELWAIN, T.J. & LINDSAY, K.S. (1988). Semen cryopreservation for patients surviving malignant disease: Implications of proposed legislation. *Lancet*, **ii**, 1197.

SUTCLIFFE, S.B. (1987). Infertility and gonadal function in Hodgkin's disease. In *Hodgkin's Disease*, Selby, P.J. & McElwain, T.J. p. 339. Blackwell Scientific Publications: Oxford.

WAXMAN, J. (1985). Cancer, chemotherapy and fertility. *Br. Med. J.*, **290**, 1096.


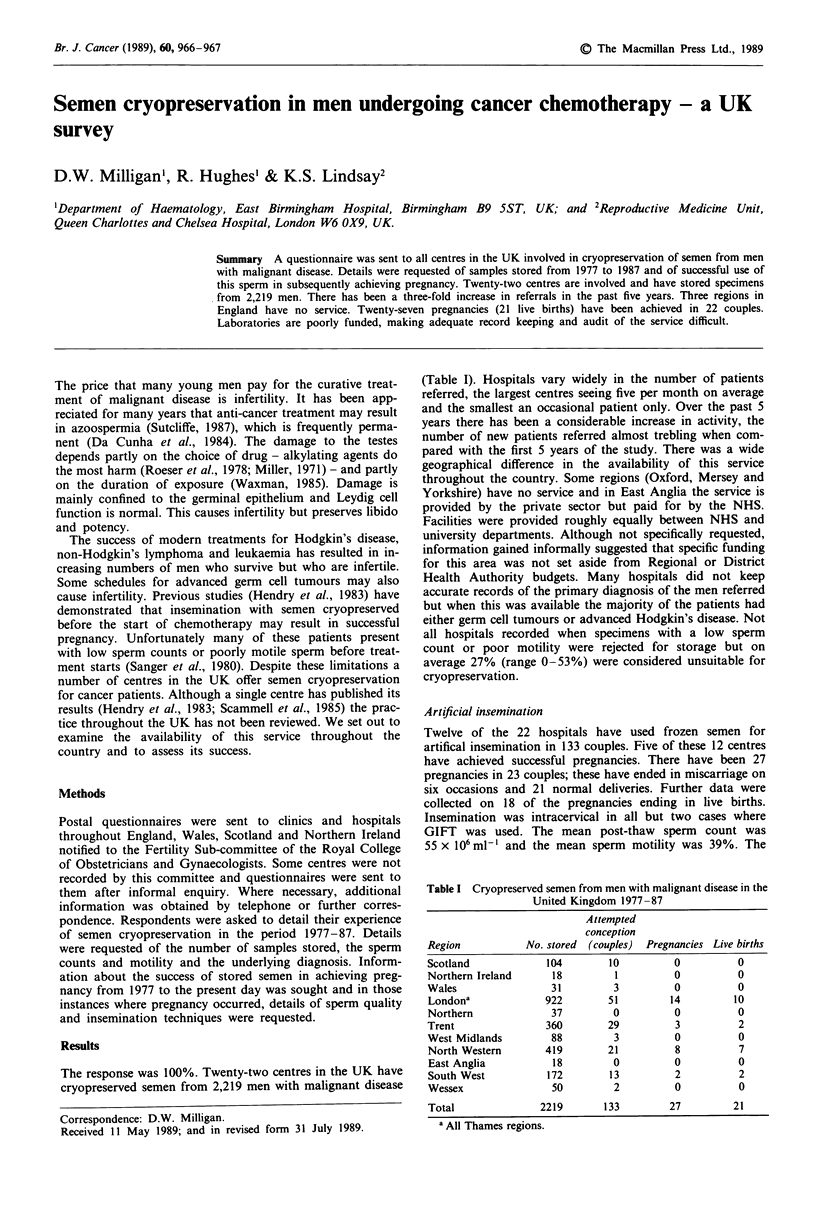

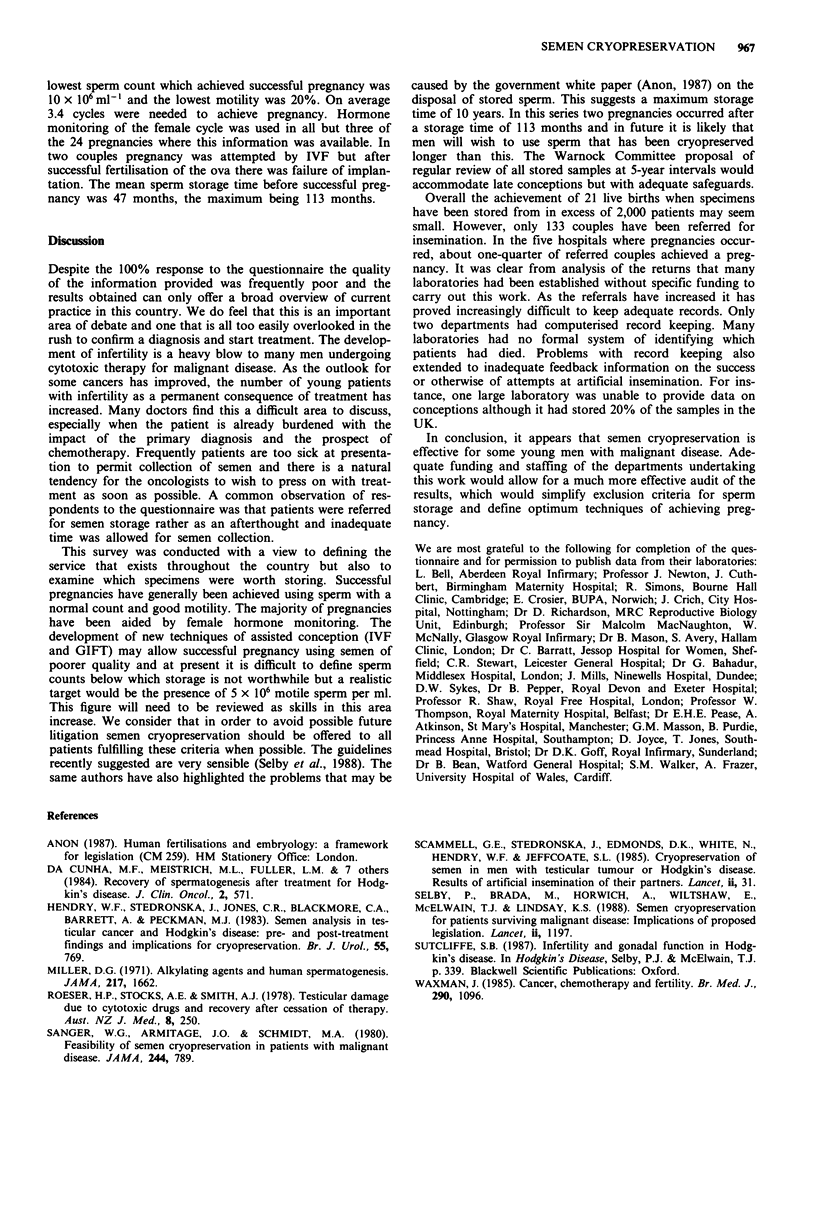


## References

[OCR_00255] Hendry W. F., Stedronska J., Jones C. R., Blackmore C. A., Barrett A., Peckham M. J. (1983). Semen analysis in testicular cancer and Hodgkin's disease: pre- and post-treatment findings and implications for cryopreservation.. Br J Urol.

[OCR_00262] Miller D. G. (1971). Alkylating agents and human spermatogenesis.. JAMA.

[OCR_00266] Roeser H. P., Stocks A. E., Smith A. J. (1978). Testicular damage due to cytotoxic drugs and recovery after cessation of therapy.. Aust N Z J Med.

[OCR_00271] Sanger W. G., Armitage J. O., Schmidt M. A. (1980). Feasibility of semen cryopreservation in patients with malignant disease.. JAMA.

[OCR_00276] Scammell G. E., White N., Stedronska J., Hendry W. F., Edmonds D. K., Jeffcoate S. L. (1985). Cryopreservation of semen in men with testicular tumour or Hodgkin's disease: results of artificial insemination of their partners.. Lancet.

[OCR_00281] Selby P., Brada M., Horwich A., Wiltshaw E., McElwain T. J., Lindsay K. S. (1988). Semen cryopreservation for patients surviving malignant disease: implications of proposed legislation.. Lancet.

[OCR_00292] Waxman J. (1985). Cancer, chemotherapy, and fertility.. Br Med J (Clin Res Ed).

[OCR_00250] da Cunha M. F., Meistrich M. L., Fuller L. M., Cundiff J. H., Hagemeister F. B., Velasquez W. S., McLaughlin P., Riggs S. A., Cabanillas F. F., Salvador P. G. (1984). Recovery of spermatogenesis after treatment for Hodgkin's disease: limiting dose of MOPP chemotherapy.. J Clin Oncol.

